# Behavioral intention to use mental health chatbots in Saudi Arabia: a cross-sectional UTAUT-based study

**DOI:** 10.3389/fpubh.2026.1926484

**Published:** 2026-09-03

**Authors:** Zaenb Alsalman, Abdullah Almaqhawi, Abdullah Almulhim, Anwar Fawzi Alqadeep, Ghadeer Mohammed Almutawah, Fatemah Ali Al Khalifa, Fatima Ramzi Alghadeer, Hawra Adnan Aleid, Walaa Mohammed Alkhalifah, Zainab Abdrabulridha Alabdullah, Safiah Ahmed AlAmer, Marwa Mahmoud Shafey

**Affiliations:** 1 Department of Family and Community Medicine, College of Medicine, King Faisal University, Al-Ahsa, Saudi Arabia; 2 College of Medicine, King Faisal University, Al-Ahsa, Saudi Arabia; 3 Family Medicine Academy, Al-Ahsa, Saudi Arabia; 4 Department of Family and Community Medicine, College of Medicine, Imam Abdulrahman bin Faisal University, Dammam, Saudi Arabia

**Keywords:** artificial intelligence, mental health chatbots, privacy risk, Saudi Arabia, stigma, technology acceptance, treatment gap, UTAUT model

## Abstract

**Background and objectives:**

Despite the growing burden of mental health conditions in Saudi Arabia, sociocultural stigma, privacy concerns, and structural barriers leave a substantial proportion of the population with unmet psychological needs. Mental health chatbots offer an anonymous, accessible, and low-barrier alternative to traditional care, aligning with the Kingdom’s digital health transformation goals. This study investigated factors associated with behavioral intention to use mental health chatbots among adults in Saudi Arabia.

**Materials and methods:**

A cross-sectional online survey was conducted among adults in Saudi Arabia. Data were collected using a questionnaire that assessed sociodemographic characteristics, mental health history, prior use of chatbots, technology acceptance based on a modified Unified Theory of Acceptance and Use of Technology (UTAUT) framework, and perceived barriers.

**Results:**

Among 533 participants (mean age 30.5 ± 11.7 years), 35.3% reported current psychological symptoms, and 51.6% reported needing psychological support during the previous year. Overall, 59.0% supported the integration of mental health chatbots into psychological services. In the primary multivariable linear regression analysis, perceived usefulness, perceived ease of use, social influence, and perceived need for psychological consultation were independently associated with higher behavioral intention.

**Conclusion:**

Behavioral intention to use mental health chatbots was primarily associated with perceived usefulness, perceived ease of use, and social influence. Perceived self-efficacy, perceived barriers, and most demographic characteristics were not independently associated with behavioral intention. These findings may inform the design and future evaluation of culturally appropriate mental health chatbot interventions in Saudi Arabia, although further research is needed to assess their real-world effectiveness and safety.

## 1 Introduction

Mental health disorders represent a major and growing global public health challenge, contributing substantially to the worldwide burden of disease (1, 2). Despite increasing recognition of this burden and rising demand for care following the COVID-19 pandemic, access to psychiatric and psychological services remains constrained by underinvestment, workforce shortages, financial barriers, and geographic disparities (3). In Saudi Arabia, despite substantial government efforts to expand healthcare infrastructure, societal stigma and cultural attitudes continue to hinder formal help-seeking behaviors (4).

To address these gaps in care, digital health technologies have become increasingly integrated into healthcare systems, offering scalable, affordable, and personalized support while expanding treatment options for both patients and clinicians (5). At the same time, the rapid development of generative artificial intelligence (AI) and large language models, such as ChatGPT, Claude, and Gemini, has increased public interest in AI-based conversational tools for mental health support (6). These general-purpose conversational AI systems differ from purpose-built mental health chatbots, which are specifically designed to provide psychological support and may offer functions such as psychoeducation, guided emotional support, mindfulness exercises, and cognitive behavioral therapy-based techniques, although their capabilities vary across platforms (7). Evidence suggests that mental health chatbots can reduce symptoms of depression, anxiety, and psychological distress, with some studies reporting outcomes comparable to those of traditional low-intensity interventions (8). By providing on-demand, smartphone-based support, these tools help overcome logistical and financial barriers to care, while their inherent anonymity may reduce concerns related to mental health stigma (3).

The effectiveness of digital mental health interventions depends largely on user acceptance (5, 8). Negative attitudes, limited trust, and low digital literacy can reduce engagement and contribute to digital exclusion (5). In the context of AI-driven tools, intention of use may also be influenced by concerns regarding privacy, transparency, and data handling. When users are uncertain about how sensitive information is collected, stored, or used, their willingness to disclose personal health information may decline (9). Therefore, theory-based frameworks are needed to better understand the factors associated with more adoption. The Unified Theory of Acceptance and Use of Technology (UTAUT), developed by Venkatesh et al., is one of the most widely used frameworks for evaluating technology acceptance in healthcare (10). Previous studies using the UTAUT framework have consistently identified performance expectancy [perceived usefulness (PU)], effort expectancy [perceived ease of use (PEOU)], and social influence as key predictors of behavioral intention (BI) to adopt digital mental health technologies (5, 8, 11–13). More recent evidence has also identified technology self-efficacy, conceptualized in this study as perceived self-efficacy (PSE), as an important predictor of healthcare consumers’ intention to adopt digital health technologies (14). However, findings regarding privacy concerns and perceived barriers have been inconsistent. While international studies have examined acceptance of mental health chatbots, these findings may not be directly generalizable to Saudi Arabia because cultural factors, including social norms, mental health stigma, and characteristics of the healthcare system, may influence technology acceptance (4, 5, 8, 11–13).

Moreover, evidence from the Arab region is largely limited to studies involving university students or evaluating broader digital mental health or psychological health applications rather than mental health chatbots specifically (4, 15, 16). Understanding public acceptance among adults in Saudi Arabia is therefore essential to inform policymakers and clinicians and support the safe, ethical, and effective integration of AI-assisted mental health services in line with Saudi Vision 2030.

Accordingly, this study investigated factors associated with BI to use mental health chatbots among adults in Saudi Arabia using a modified UTAUT framework. Based on this framework, the following hypotheses were proposed:

*H1*: Higher perceived usefulness is positively associated with BI to use mental health chatbots.

*H2*: Higher perceived ease of use is positively associated with BI to use mental health chatbots.

*H3*: Higher perceived social influence is positively associated with BI to use mental health chatbots.

*H4*: Higher perceived self-efficacy is positively associated with BI to use mental health chatbots.

*H5*: Higher perceived barriers (including privacy, data security, and human-equivalence concerns) are negatively associated with BI to use mental health chatbots.

## 2 Materials and methods

### 2.1 Study design and participants

A cross-sectional study was conducted between August and December 2025 to examine factors associated with BI to use mental health chatbots among Saudi adults. Participants were recruited through an online self-administered questionnaire distributed via social media platforms and regional community networks using a convenience sampling strategy. Eligible participants were adults aged 18 years or older, living in Saudi Arabia, and able to read and understand Arabic. Participation was voluntary, and electronic informed consent was obtained by asking participants to confirm their consent before accessing the questionnaire. The minimum required sample size was calculated using OpenEpi. Assuming a 95% confidence level, a 5% margin of error, and a conservative expected proportion of 50% for technology adoption, the required sample size was 385 participants. This calculation was performed to estimate the sample required for the cross-sectional survey and was not intended to determine the sample size for the multivariable regression analyses. Only complete questionnaires were analyzed, and responses were limited to one submission per device. No formal attention-check questions were included in the questionnaire.

### 2.2 Data collection

Data were collected using a self-administered Arabic-language questionnaire developed specifically for this study and informed by the literature on technology acceptance, digital mental health interventions, and the Unified Theory of Acceptance and Use of Technology (UTAUT) framework (4, 5, 10, 12, 14). The Arabic and English versions of the questionnaire are provided in Supplementary data 1. The research team reviewed questionnaire to ensure its relevance and clarity, and a pilot test involving 20 participants confirmed its readability. The survey instrument consisted of five sections: Section 1: Sociodemographic Characteristics: including age, gender, educational level, and employment status. Section 2: Mental Health Background: evaluated respondents’ personal mental health context over the preceding 12 months, including current psychological symptoms, history of formal mental health diagnoses, history of psychological treatment, and perceived need for professional psychological support. Section 3: Prior Use of Chatbots: assessed past use of chatbots for mental health-related purposes and supported integrating these chatbots into formal Saudi mental health services. Participants were asked to consider chatbots used for psychological support or mental health information. No specific applications were named, and the questionnaire did not require participants to distinguish between general-purpose conversational AI and dedicated mental health chatbots. Section 4: Technology Acceptance Constructs (Modified UTAUT Framework): assessed acceptance of mental health chatbots. The original UTAUT framework comprises four core constructs: Performance Expectancy, Effort Expectancy, Social Influence, and Facilitating Conditions (10). The questionnaire operationalized the modified UTAUT constructs in the context of mental health chatbot use while maintaining consistency with the underlying theoretical concepts. In this study, Performance Expectancy was operationalized as Perceived Usefulness (PU), using context-specific items to assess the perceived benefits of mental health chatbot use. Effort Expectancy was operationalized as Perceived Ease of Use (PEOU) using items reflecting the ease of learning and using mental health chatbots. Social Influence (SI) was retained as a construct, referring to the perceived influence of family, friends, and society on participants’ intention to use mental health chatbots. Facilitating Conditions were replaced with Perceived Self-Efficacy (PSE), defined as participants’ confidence in accessing and using mental health chatbots, while recognizing that some items also reflect the perceived availability of support when difficulties arise. This modification was made because mental health chatbots are typically accessed through personal smartphones or web-based platforms rather than within organizational settings supported by formal technical infrastructure. Therefore, technology self-efficacy was considered more relevant to BI than organizational facilitating conditions in the context of this study. This approach is also supported by recent evidence identifying technology self-efficacy as an important predictor of digital health technology adoption (14). These constructs were assessed using 10 items, including three items each for PU and PEOU and two items each for SI and PSE, rated on a 5-point Likert scale ranging from 1 (strongly disagree) to 5 (strongly agree). Section 5: Perceived Barriers (PB): examined participants’ concerns regarding the use of mental health chatbots, including privacy and data security issues, doubts about the quality of mental health chatbots support relative to human therapists, and concerns about becoming overly dependent on such tools. This construct was measured using four items rated on a 5-point Likert scale ranging from 1 (strongly disagree) to 5 (strongly agree). Item scores were summed to generate a cumulative PB score, with higher scores indicating greater perceived barriers. The subscales demonstrated moderate to excellent reliability, with Cronbach’s alpha values of 0.888 for PU, 0.764 for PEOU, 0.861 for BI, and 0.805 for PB. For the two-item constructs, SI and PSE, internal consistency was assessed using the Pearson inter-item correlation and Spearman–Brown coefficient. The SI scale demonstrated an inter-item correlation of 0.437 and a Spearman–Brown coefficient of 0.608, whereas PSE demonstrated an inter-item correlation of 0.551 and a Spearman–Brown coefficient of 0.710.

**Figure 1:**
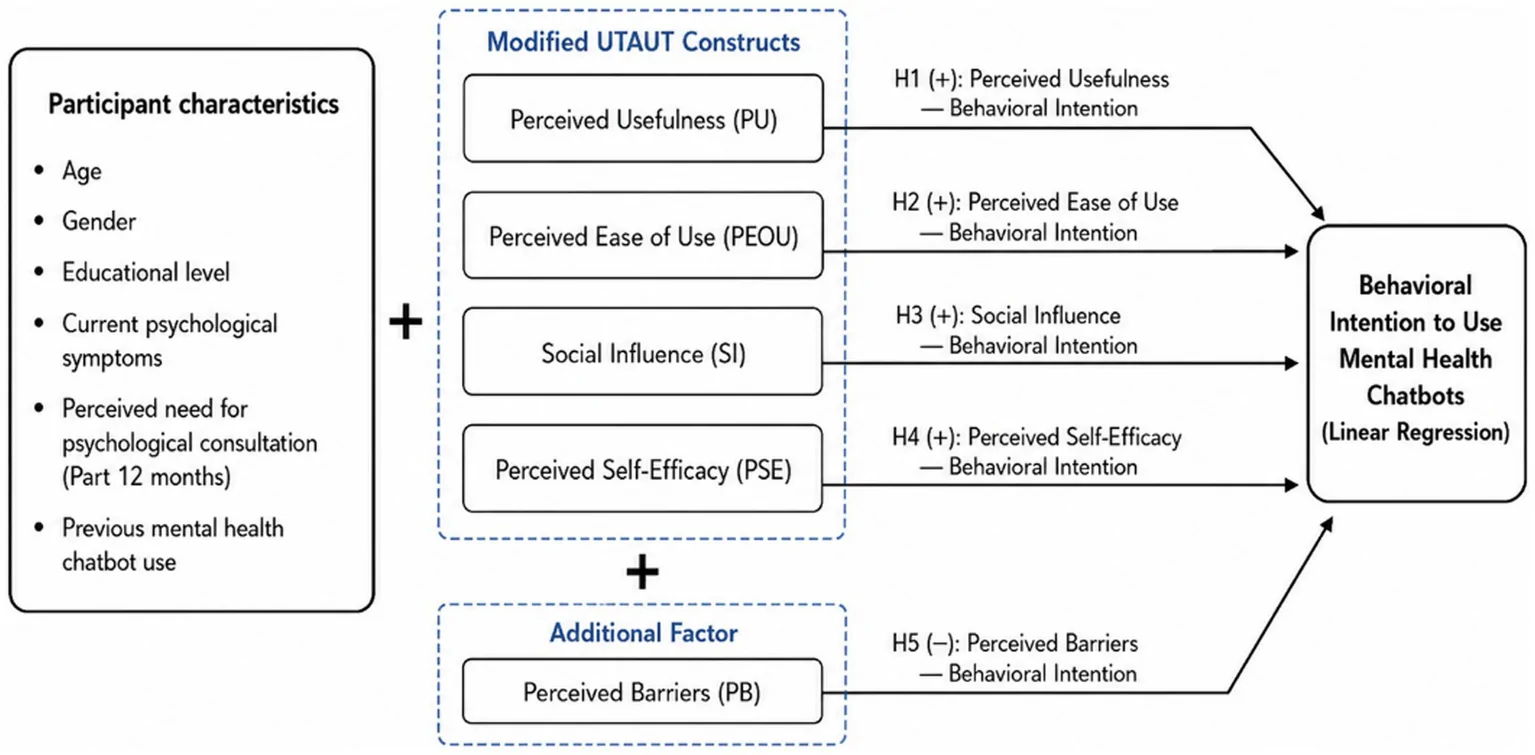
Conceptual framework illustrating the hypothesized relationships between the modified UTAUT constructs, additional factors, and behavioral intention to use mental health chatbots. Participants’ characteristics (e.g., gender, educational level, current psychological symptoms, perceived need for psychological consultation during the past 12 months, and previous mental health chatbot use) were included as covariates in the multivariable linear regression analysis.

Based on the modified UTAUT framework, a conceptual framework was developed to illustrate the hypothesized relationships between the modified UTAUT constructs, PB, and BI to use mental health chatbots (Figure 1).

### 2.3 Statistical analysis

Data were analyzed using SPSS software version 28. Categorical variables were summarized as frequencies and percentages, while continuous variables were presented as means and standard deviations (SD).

For each construct, item scores were summed to generate cumulative scale scores, with higher scores indicating higher levels of the corresponding construct. BI, consisting of two items, yielded a continuous score ranging from 2 to 10. Multivariable linear regression with the continuous BI score was the primary analysis, with age, gender, education, current psychological symptoms, perceived need for psychological consultation or support during the preceding 12 months, previous mental health chatbot use, PU, PEOU, SI, PSE, and PB included as predictors. Regression coefficients (B), standard errors (SE), 95% confidence intervals (CIs), and *p*-values were reported.

For descriptive and supplementary categorical analyses, BI was categorized as low (≤5) and high (>5). PU and PEOU were categorized as low (<8) and high (≥8), SI and PSE as low (≤5) and high (>5), and PB as low (<10) and high (≥10), using a pragmatic threshold. These categorical definitions were used only for descriptive and supplementary analyses. A supplementary multivariable binary logistic regression analysis was performed to examine factors associated with high BI (BI >5), with adjusted odds ratios (AORs) and 95% confidence intervals (CIs) reported. Multicollinearity was assessed using variance inflation factors (VIFs) and tolerance values. A two-sided *p*-value <0.05 was considered statistically significant.

## 3 Results

### 3.1 Sociodemographic and mental health–related characteristics of participants

A total of 533 participants were included (mean age 30.45 ± 11.71 years). Most participants were female (80.1%), aged 21–30 years (45.2%), and held a bachelor’s degree (68.1%). Students represented the largest occupational group (41.7%). More than one-third of participants (35.3%) reported current psychological symptoms, while 8.6% reported a previous mental health diagnosis. Only 3.2% were currently receiving psychological treatment. Overall, 51.6% of participants reported needing psychological consultation or mental health support during the previous 12 months (Table 1).

**Table 1:** Sociodemographic characteristics, mental health status, and mental health chatbot use among participants (*n* = 533).

Variable	*n* = 533	%
Age		
18–20	84	15.8
21–30	241	45.2
31–40	94	17.6
41–50	81	15.2
>50	33	6.2
Gender		
Male	106	19.9
Female	427	80.1
Educational level		
Secondary school or below	107	20.1
Diploma	35	6.6
Bachelor’s degree	363	68.1
Postgraduate studies	28	5.2
Employment status		
Employee in the mental health sector	1	0.2
Employee in the information technology sector	11	2.1
Employee in another sector	195	36.5
Student	222	41.7
Unemployed	104	19.5
Are you currently experiencing psychological symptoms (such as anxiety, depression, stress, or others)?		
Yes	188	35.3
No	345	64.7
Have you ever been diagnosed with a mental disorder by a specialist?		
Yes	46	8.6
No	487	91.4
Have you ever received any psychological treatment from a specialist (pharmacological or behavioral)?		
Yes, I am currently receiving treatment	17	3.2
Yes, I have received treatment in the past	36	6.7
No, I have never received psychological treatment	480	90.1
During the past 12 months, have you felt that you needed psychological consultation or mental health support?		
Yes	275	51.6
No	258	48.4
Have you ever used an intelligent chatbot for psychological purposes (such as emotional support or seeking mental health information)?		
Yes	232	43.5
No	301	56.5
Do you support integrating mental health chatbots into mental health services?		
Yes	315	59
No	218	41
Behavioral intention (BI)		
Low (≤5)	166	31.1
High (>5)	367	68.9

### 3.2 Use of chatbots for mental health support

Approximately 43.5% reported having previously used chatbots for psychological support or mental health information. The majority of participants (59%) supported integrating chatbots into mental health services. Regarding behavioral intention, 367 (68.9%) participants had high BI (BI >5), while 166 (31.1%) had low BI (BI ≤5) (Table 1).

### 3.3 Participants’ responses across the modified UTAUT and perceived barriers domains

Participants’ responses toward mental health chatbot utilization across different domains are presented in Table 2.

**Table 2:** Participants’ responses across the modified UTAUT and perceived barriers domains (*n* = 533).

Variable	Strongly disagree *n* (%)	Disagree *n* (%)	Neither agree nor disagree *n* (%)	Agree *n* (%)	Strongly agree *n* (%)
Perceived usefulness (PU)	Mean (SD): 8.66 (3.23)				
1- I believe that using a chatbot can improve my psychological well-being.	89 (16.6)	81 (15.2)	205 (38.5)	116 (21.8)	42 (7.9)
2- The chatbot helps me manage my emotions better.	106 (19.8)	99 (18.6)	173 (32.5)	117 (22.0)	38 (7.1)
3- The chatbot may contribute to reducing my stress or anxiety.	85 (15.9)	89 (16.7)	155 (29.1)	152 (28.5)	52 (9.8)
Perceived ease of use (PEOU)	Mean (SD): 10.50 (3.08)				
4- It is easy for me to learn how to use artificial intelligence chat applications for psychological support.	64 (12.0)	57 (10.6)	139 (26.1)	173 (32.5)	100 (18.8)
5- Using a chatbot does not require extensive technical expertise.	47 (8.8)	43 (8.1)	83 (15.6)	167 (31.3)	193 (36.2)
6- I can easily interact with a chatbot that provides psychological services.	57 (10.6)	60 (11.3)	161 (30.2)	141 (26.5)	114 (21.4)
Perceived social influence (SI)	Mean (SD): 5.56 (2.05)				
7- People around me encourage me to try psychological support chatbots.	164 (30.8)	128 (24.0)	143 (26.8)	56 (10.5)	42 (7.9)
8- I believe that society has begun to accept the use of artificial intelligence chat applications in mental health.	61 (11.4)	80 (15.0)	174 (32.6)	150 (28.2)	68 (12.8)
Perceived self-efficacy (PSE)	Mean (SD): 6.75 (2.16)				
9- I have the ability to access an application or website that includes a mental health chatbot.	63 (11.8)	64 (12.0)	128 (24.0)	170 (31.9)	108 (20.3)
10- I can obtain assistance if I encounter difficulty in using the chatbot.	50 (9.4)	59 (11.1)	161 (30.2)	161 (30.2)	102 (19.1)
Perceived barriers (PB)	Mean (SD): 10.68 (4.18)				
1- I feel concerned about sharing my personal feelings with a chatbot.	100 (18.8)	107 (20.1)	148 (27.7)	84 (15.8)	94 (17.6)
2- I am afraid that my psychological data may be used in an unsafe manner when using a chatbot.	65 (12.2)	83 (15.6)	107 (20.1)	126 (23.6)	152 (28.5)
3- I believe that chatbots may not provide psychological support with the same quality as a human therapist.	47 (8.9)	58 (10.9)	134 (25.1)	135 (25.3)	159 (29.8)
4- I am concerned about over-reliance on chatbots instead of traditional therapy.	60 (11.3)	58 (10.8)	152 (28.5)	125 (23.5)	138 (25.9)
Behavioral intention to use (BI)	Mean (SD): 6.34 (2.46)				
1- If a reliable chatbot that provides psychological support is available, I will use it.	75 (14.1)	53 (9.9)	136 (25.5)	156 (29.3)	113 (21.2)
2- I intend to use an intelligent chatbot to obtain psychological support when needed.	109 (20.5)	64 (12.0)	151 (28.3)	135 (25.3)	74 (13.9)

The mean score for PU was 8.66 ± 3.23. Regarding improvement of psychological well-being, 38.5% of participants reported a neutral response, while 21.8% agreed and 7.9% strongly agreed with the statement. Concerning emotional management, responses were mainly neutral (32.5%), whereas 22.0% agreed and 7.1% strongly agreed that chatbots help manage emotions better. Similarly, 29.1% of participants expressed neutrality toward the role of chatbots in reducing stress or anxiety, with 28.5% agreeing and 9.8% strongly agreeing.

PEOU demonstrated a mean score (10.50 ± 3.08). About one-third of participants (32.5%) agreed and 18.8% strongly agreed that learning to use chatbot for psychological support is easy. Regarding technical complexity, 31.3% agreed and 36.2% strongly agreed that chatbot use does not require extensive technical expertise. Additionally, 30.2% reported neutral responses toward ease of interaction with mental health chatbots, while 26.5% agreed and 21.4% strongly agreed.

The mean score for perceived SI was 5.56 ± 2.05. Nearly one-third of participants (30.8%) strongly disagreed that people around them encourage mental health chatbot use, while 26.8% reported neutral perceptions. In contrast, response toward societal acceptance were more favourable, with 32.6% reporting neutrality, 28.2% agreement, and 12.8% strong agreement.

Regarding PSE participants showed confidence in their ability to use chatbot, with a mean score of 6.75 ± 2.16. Approximately 31.9% agreed and 20.3% strongly agreed that they have access to applications or websites containing mental health chatbots. Furthermore, 30.2% agreed and 19.1% strongly agreed that assistance would be available if difficulties were encountered during chatbot use.

Concerns regarding chatbot utilization were evident across several items. About 27.7% of participants reported neutral responses toward sharing personal feelings with chatbots, while 17.6% strongly agreed with this concern. Fear related to unsafe use of psychological data was reported by 23.6% who agreed and 28.5% who strongly agreed. Additionally, 25.3% agreed and 29.8% strongly agreed that chatbots may not provide psychological support comparable to human therapists. Concerns regarding over-reliance on chatbots instead of traditional therapy were also observed, with 23.5% agreeing and 25.9% strongly agreeing.

The mean BI score was 6.34 ± 2.46. Regarding willingness to use a reliable chatbot, 29.3% agreed and 21.2% strongly agreed, while 25.5% remained neutral. Similarly, 28.3% expressed neutrality toward intending to use chatbots when psychological support is needed, whereas 25.3% agreed and 13.9% strongly agreed.

### 3.4 Factors associated with BI to use mental health chatbots

No significant differences in BI were observed across age (Kruskal–Wallis *p* = 0.828; Spearman *r* = 0.043, *p* = 0.318), gender (*p* = 0.501), or educational level (*p* = 0.616). Similarly, no significant differences were observed according to a previous diagnosis of mental disorders (*p* = 0.846) or history of psychological treatment (*p* = 0.605). In contrast, mental health status was significantly associated with BI. Participants reporting current psychological symptoms showed higher BI scores than those without symptoms (Z = −2.763, *p* = 0.006). Likewise, those who perceived a need for psychological consultation or support during the previous 12 months demonstrated significantly higher BI (Z = −5.311, *p* < 0.001). Prior use of chatbots for psychological purposes was also associated with higher BI (Z = −5.619, *p* < 0.001).

Regarding the modified UTAUT constructs, all positive attitudinal domains were significantly associated with higher BI. Specifically, higher PU (Z = −10.808, *p* < 0.001), PEOU (Z = −10.380, *p* < 0.001), SI (Z = −10.069, *p* < 0.001), and PSE (Z = −8.920, *p* < 0.001) were all associated with higher BI. In contrast, PB was not significantly associated with BI (*p* = 0.916) (Table 3).

**Table 3:** Comparison of behavioral intention scores across participant characteristics (*n* = 533).

Variable	Behavioral intention (BI), mean (SD) 6.34 ± 2.46	Behavioral intention (BI), median (IQR) 6 (5–8)	Test statistic, *p*-value
Age			1.492 (*p* = 0.828)^£^
18–20	6.42 (2.53)	7 (4–8)	
21–30	6.17 (2.52)	6 (4–8)	
31–40	6.48 (2.42)	6 (5–8)	
41–50	6.50 (2.31)	6 (6–8)	
>50	6.52 (2.36)	7 (5–8)	
Gender			
Male	6.52 (2.30)	6.5 (5–8)	−0.673 (*p* = 0.501)^$^
Female	6.29 (2.50)	6 (4–8)	
Educational level			1.796 (*p* = 0.616)^£^
Secondary school or below	6.14 (2.53)	6 (4–8)	
Diploma	6.00 (2.74)	6 (4–8)	
Bachelor’s degree	6.39 (2.44)	7 (5–8)	
Postgraduate studies	6.82 (2.00)	6 (6–8)	
Are you currently experiencing psychological symptoms (such as anxiety, depression, stress, or others)?			−2.763 (*p* = 0.006)^$^
Yes	6.75 (2.32)	7 (5–8)	
No	6.11 (2.50)	6 (6–8)	
Have you ever been diagnosed with a mental disorder by a specialist?			−0.194 (*p* = 0.846)^$^
Yes	6.37 (2.64)	6.5 (4.75–8)	
No	6.33 (2.44)	6 (5–8)	
Have you ever received psychological treatment from a specialist (pharmacological or behavioral)?			1.006 (*p* = 0.605)^£^
Yes, I am currently receiving treatment	6.29 (2.37)	6 (5–8.5)	
Yes, I received treatment in the past only	6.72 (2.43)	7 (5.25–8)	
No, I have never received psychological treatment	6.31 (2.47)	6 (4–8)	
During the past 12 months, have you felt that you needed psychological consultation or mental health support?			−5.311 (*p* < 0.001)^$^
Yes	6.87 (2.36)	7 (6–8)	
No	5.77 (2.44)	6 (4–8)	
Have you ever used chatbots for psychological purposes (such as emotional support or seeking mental health information)?			−5.619 (*p* < 0.001)^$^
Yes	7.00 (2.30)	8 (6–9)	
No	5.82 (2.45)	6 (4–8)	
Perceived usefulness (PU)			−10.808 (*p* < 0.001)^$^
Low<8	4.67 (2.38)	4 (2–6.5)	
High≥8	7.20 (2.02)	8 (6–8.75)	
Perceived ease of use (PEOU)			−10.380 (*p* < 0.001)^$^
Low<8	3.76 (2.16)	3 (2–5)	
High≥8	6.88 (2.16)	7 (6–8)	
Social influence (SI)			−10.069 (*p* < 0.001)^$^
Low≤5	5.22 (2.45)	5 (3–7)	
High>5	7.38 (1.96)	8 (6–9)	
Perceived self-efficacy (PSE)			−8.920 (*p* < 0.001)^$^
Low≤5	4.67 (2.40)	4 (2–6)	
High>5	6.91 (2.21)	7 (6–8)	
Perceived barriers (PB)			−0.106 (*p* = 0.916)^$^
Low<10	6.37 (2.40)	7 (4–8)	
High≥10	6.30 (2.54)	6 (5–8)	

*Test of significance; $: Mann Whitney, £: Kruskal–Wallis tests.

In the primary multivariable linear regression analysis, higher PU (B = 0.263, 95% CI 0.199–0.326, *p* < 0.001), PEOU (B = 0.186, 95% CI 0.113–0.259, *p* < 0.001), and SI (B = 0.248, 95% CI 0.160–0.336, *p* < 0.001) were significantly and positively associated with BI. Perceived need for psychological consultation or support was also positively associated with BI (B = 0.437, 95% CI 0.093–0.780, *p* = 0.013), as was age, although the association was small (B = 0.016, 95% CI 0.002–0.031, *p* = 0.030). PSE (B = 0.068, *p* = 0.158) and PB (B = 0.009, *p* = 0.635) were not significantly associated with BI (Table 4). Multicollinearity diagnostics indicated no evidence of problematic multicollinearity among the predictors, with tolerance values ranging from 0.426 to 0.934 and VIF values ranging from 1.070 to 2.345. A supplementary binary logistic regression analysis using high BI (BI >5) as the outcome is presented in Supplementary Table S1.

**Table 4:** Multivariable linear regression analysis of factors associated with behavioral intention to use mental health chatbots.

Predictor	B	SE	95% CI for B	*p*-value
Age	0.016	0.008	0.002–0.031	0.030
Gender	−0.134	0.201	−0.528 to 0.260	0.504
Education	−0.035	0.093	−0.218 to 0.147	0.704
Current psychological symptoms	0.141	0.172	−0.197 to 0.480	0.412
Needed psychological consultation/support	0.437	0.175	0.093–0.780	0.013
Ever used a mental health chatbot	0.148	0.175	−0.197 to 0.492	0.401
Perceived usefulness (PU)	0.263	0.032	0.199–0.326	<0.001
Perceived ease of use (PEOU)	0.186	0.037	0.113–0.259	<0.001
Social influence (SI)	0.248	0.045	0.160–0.336	<0.001
Perceived self-efficacy (PSE)	0.068	0.048	−0.026–0.161	0.158
Perceived barriers (PB)	0.009	0.018	−0.028 to 0.045	0.635

## 4 Discussion

The growing demand for accessible mental health support has increased interest in AI-based mental health technologies. While previous studies in Saudi Arabia have indicated that the public holds favorable opinions of AI in healthcare and have largely focused on conventional digital mental health applications such as Labayh, our study extends this evidence by examining adults’ acceptance of AI-driven conversational systems (4, 17). The findings indicate a generally positive response toward mental health chatbots, with BI scores suggesting moderate willingness to use these tools and most participants supporting their integration into mental health services. This suggests that mental health chatbots may be viewed as a valuable addition to existing mental health resources. Acceptance was primarily associated with PU, PEOU, and SI. Participants reporting a perceived need for professional consultation had higher BI scores. Among demographic characteristics, increasing age showed a modest independent association with BI, whereas gender and educational level were not associated with chatbot acceptance.

Consistent with prior research on digital health and technology adoption, younger adults and individuals with at least a bachelor’s degree were overrepresented in our sample. This pattern likely reflects their greater exposure to and familiarity with digital platforms, as well as a higher likelihood of adopting new health technologies (4). Despite more than half of participants reporting a need for psychological consultation in the past 12 months and over one-third reporting psychological symptoms, only 3.2% were actively receiving treatment. This marked gap between mental health needs and treatment uptake aligns with evidence from Saudi Arabia and other countries showing that many individuals with psychiatric symptoms remain untreated (18–21). However, because these measures reflect different timeframes, the apparent gap should be interpreted with caution.

The moderate behavioral intention and broad support for integrating mental health chatbots into mental health services suggest that these tools may offer an accessible option for expanding mental health support, consistent with previous evidence (22). In the current study, BI was not associated with most demographic or clinical characteristics, including gender, educational level, previous mental health diagnosis, or previous treatment. Although age was not associated with BI in the bivariate analysis, it showed a modest independent association in the multivariable model. This finding is consistent with recent studies suggesting that attitudinal factors may play a greater role in BI to use mental health chatbots than demographic characteristics. Naji et al. reported that attitudes toward mental health chatbots were stronger predictors of BI than baseline demographics, while ElBarazi et al. found that socio-demographic and clinical factors became non-significant after accounting for trust, confidentiality, and perceived platform compassion (15, 23). In addition, participants who reported a perceived need for professional consultation within the past year had higher BI. This may suggest that individuals who recognize a need for mental health support are more willing to use mental health chatbots, possibly because these tools are perceived as accessible and timely sources of support that may reduce barriers to seeking care (24, 25).

Similar to findings among Saudi users of the Labayh application, PU showed a strong positive association with BI (4). This suggests that perceiving clear psychological benefits may be associated with greater willingness to use mental health chatbots, although actual use was not evaluated in this study. However, greater PU does not necessarily imply a preference for fully autonomous care. Previous studies suggest that AI-based mental health tools may complement rather than replace mental health professionals, emphasizing the im.portance of human oversight in psychological care (6). Accordingly, communicating the potential benefits of mental health chatbots while emphasizing their complementary role may support their acceptance within existing mental health services.

In contrast to Li et al., who observed an inverse relationship and suggested that overly simple interfaces might signal a lack of clinical sophistication, PEOU was positively associated with BI in our study (8). This finding may reflect participants’ familiarity with digital technologies, as recent local surveys suggest that widespread exposure to conversational systems such as ChatGPT may have increased comfort with intuitive digital interfaces, potentially facilitating acceptance of user-friendly mental health chatbots (17). Social influence was also positively associated with BI, consistent with the extended UTAUT framework proposed by Békés et al. (5). This may reflect the role of family and social networks in shaping health-related decisions in Saudi Arabia and is consistent with previous studies from the Gulf region (4, 11, 16). Public awareness campaigns and endorsement by healthcare professionals may therefore further support acceptance.

Although greater PSE was associated with higher BI in the bivariate analysis, this association was not statistically significant in the primary multivariable linear regression. Previous research has identified self-efficacy as a relevant factor in digital health technology acceptance; however, these studies examined broader digital health technologies rather than mental health chatbots specifically (14, 16). In our study, PSE was intended to capture confidence in accessing and using mental health chatbots, although some items may also reflect aspects of facilitating conditions. Therefore, the role of PSE in BI remains uncertain and warrants further investigation using more clearly defined measures.

Additionally, prior use of chatbots for psychological purposes was associated with higher BI in the bivariate analysis but was not independently associated with BI in the multivariable model. This may indicate that the association between previous chatbot use and BI is partly accounted for by factors such as perceived usefulness, ease of use, and social influence. This interpretation is consistent with Yang et al., who noted that although past digital interactions may increase familiarity, users’ perceptions of a platform’s usefulness and security may be more strongly associated with adoption (7). However, this should be interpreted cautiously, as chatbot types were not distinguished in current study.

Although concerns regarding privacy, data security, clinical quality, and over-reliance were common among participants, perceived barriers were not significantly associated with BI in the final model. This finding was somewhat unexpected, as we hypothesized a negative association between PB and BI. However, it is consistent with previous studies showing that, after accounting for key technology acceptance constructs such as PU and PEOU, privacy concerns may no longer be independently associated with intention to use mental health chatbots (7, 8). This finding should be interpreted cautiously because the composite PB score combined conceptually distinct concerns, including privacy, data security, perceived clinical quality, and dependency. Future studies should examine these domains separately using item-level or subscale analyses. Importantly, the lack of a significant association should not be interpreted as evidence that privacy and safety concerns are unimportant. Protecting personal data, ensuring transparent privacy policies, and maintaining appropriate clinical oversight remain essential for building public trust and supporting the safe use of mental health chatbots. Clear guidance on appropriate use, referral pathways for individuals experiencing severe psychological distress or suicidal ideation, clinical oversight by qualified mental health professionals, and appropriate regulatory frameworks should also be considered in future implementation strategies (5, 6, 8, 9, 18).

This study is among the first to examine BI to use mental health chatbots among adults in Saudi Arabia. These findings provide context-specific evidence that may inform the future development, evaluation, and implementation of mental health chatbots in Saudi Arabia and support ongoing digital mental health initiatives under Saudi Vision 2030. Future implementation efforts should emphasize clinical value, ease of use, and public trust while engaging healthcare professionals and other key stakeholders. Further research should evaluate whether mental health chatbots can provide a scalable, low-barrier option for individuals who may be hesitant to seek traditional mental health services.

Despite these contributions, several limitations qualify our findings. First, the cross-sectional design prevents definitive causal claims, and our reliance on self-reported data introduces potential subjective bias. Because all variables were measured using a single questionnaire at a single time point, common method bias cannot be ruled out. In addition, some overlap between perceived usefulness and behavioral intention items may have strengthened the observed associations and should be considered when interpreting the findings. Additionally, participants were recruited using convenience sampling via an online survey, resulting in a sample that was predominantly female and students. Moreover, the online survey may have introduced selection bias by attracting participants with more positive responses toward digital technologies. Therefore, the findings may not be fully representative of the broader adult population in Saudi Arabia. Furthermore, although participation was anonymous and voluntary, formal mental health referral information was not provided. This represents an ethical limitation, particularly as a proportion of participants reported current psychological symptoms. We also evaluated BI rather than actual, documented usage, as a willingness to use a chatbot does not guarantee long-term real-world engagement. The questionnaire did not distinguish between dedicated mental health chatbots and general-purpose conversational AI. Given the limited availability of dedicated Arabic-language mental health chatbots, this may have influenced participants’ responses. Regarding the psychometric evaluation, while the questionnaire demonstrated acceptable internal consistency and underwent pilot testing, formal factor analysis was omitted. Future psychometric evaluations should incorporate exploratory or confirmatory factor analysis to validate the scale structure. The relatively low reliability of the two-item Social Influence scale also warrants cautious interpretation of its association with BI. Finally, this study applied a modified UTAUT framework by operationalizing Performance Expectancy and Effort Expectancy as Perceived Usefulness and Perceived Ease of Use, and replacing Facilitating Conditions with Perceived Self-Efficacy to better reflect self-directed mental health chatbot use. As this exploratory modification was intended to examine factors associated with BI rather than validate a new framework, comparisons with studies using the original UTAUT should be made cautiously. Accordingly, the modified UTAUT framework was used as a conceptual framework rather than to test the full UTAUT model. Future research should validate this modified framework and examine the role of external facilitating factors.

## 5 Conclusion

Behavioral intention to use mental health chatbots was independently associated with perceived usefulness, perceived ease of use, social influence, and perceived need for psychological consultation or support. Perceived self-efficacy and perceived barriers were not independently associated with BI in the primary continuous analysis. These findings may inform the design and future evaluation of culturally appropriate mental health chatbot interventions in Saudi Arabia, although further research is needed to assess their real-world effectiveness and safety.

## Data Availability

The raw data supporting the conclusions of this article will be made available by the authors, without undue reservation.
