# Supplementary material for: Behavioral intention to use mental health chatbots in Saudi Arabia: a cross-sectional UTAUT-based study
Source: Front Public Health. 2026 Sep 3;14:1926484. doi: 10.3389/fpubh.2026.1926484 (PMC13582321; doi:10.3389/fpubh.2026.1926484)
Supplement: Supplementary file 1 [file Data_Sheet_1.docx]

# **تصورات المجتمع السعودي حول استخدام روبوتات الدردشة للصحة النفسية في المملكة العربية السعودية**

## ****المقدمة****

تهدف هذه الاستبانة إلى تقييم تصورات المجتمع السعودي حول استخدام **روبوتات الدردشة للصحة النفسية**.

سيستغرق إكمال هذه الاستبانة حوالي **8 دقائق**. مشاركتكم طوعية، وجميع المعلومات التي تقدمونها ستبقى سرية، ولن تُستخدم إلا لأغراض البحث العلمي.

☐ أوافق على المشاركة في هذه الدراسة.

# **القسم الأول: الخصائص الديموغرافية**

**.1 العمر:**

**.2 الجنس:**

- ☐ ذكر
- ☐ أنثى

**.3المستوى التعليمي:**

- ☐ الثانوية العامة أو أقل
- ☐ دبلوم
- ☐ البكالوريوس
- ☐ الدراسات العليا

**.4الحالة الوظيفية:**

- ☐ موظف في قطاع الصحة النفسية
- ☐ موظف في قطاع تقنية المعلومات
- ☐ موظف في قطاع آخر
- ☐ طالب/طالبة
- ☐ غير موظف

# **القسم الثاني: الخلفية المتعلقة بالصحة النفسية**

**.1هل تعاني حالياً من أعراض نفسية (مثل القلق أو الاكتئاب أو التوتر أو غيرها)؟**

- ☐ نعم
- ☐ لا

**.2 هل سبق أن شُخِّصت باضطراب نفسي من قبل أحد المختصين الصحيين؟**

- ☐ نعم
- ☐ لا

**.3هل سبق أن تلقيت علاجاً نفسياً من أحد المختصين الصحيين (دوائياً أو نفسياً)؟**

- ☐ نعم، وما زلت أتلقى العلاج
- ☐ نعم، تلقيت العلاج سابقاً فقط
- ☐ لا، لم أتلقَّ علاجاً نفسياً من قبل

**4خلال الاثني عشر شهراً الماضية، هل شعرت بالحاجة إلى استشارة نفسية؟**

- ☐ نعم
- ☐ لا

# **القسم الثالث: الاستخدام السابق لروبوتات الدردشة**

**.1هل سبق لك استخدام روبوت دردشة ذكي لأغراض نفسية، مثل الحصول على الدعم العاطفي أو المعلومات المتعلقة بالصحة النفسية؟**

- ☐ نعم
- ☐ لا

**.2هل تؤيد دمج روبوتات الدردشة للصحة النفسية ضمن خدمات الصحة النفسية في المملكة العربية السعودية؟**

- ☐ نعم
- ☐ لا

# **القسم الرابع: أبعاد تقبل التقنيه**

# يرجى تحديد مدى موافقتك على العبارات التالية.

**مقياس الإجابة:**

(1= لا أوافق بشدة /2= لا أوافق/ 3= محايد / 4= أوافق / 5= أوافق بشدة )

### ****الفائدة المتصورة****

1. أعتقد أن استخدام روبوت الدردشة يمكن أن يحسن من صحتي النفسية.
2. يساعدني روبوت الدردشة على إدارة مشاعري بشكل أفضل.
3. قد يساعد روبوت الدردشة في تقليل التوتر أو القلق لدي.

### ****سهولة الاستخدام المتصورة****

4. أجد أنه من السهل تعلم كيفية استخدام تطبيقات روبوتات الدردشة للدعم النفسي.
5. لا يتطلب استخدام روبوت الدردشة خبرة تقنية كبيرة.
6. أستطيع التفاعل بسهولة مع روبوت دردشة يقدم دعماً نفسياً.

### ****التأثير الاجتماعي****

7. يشجعني الأشخاص من حولي على تجربة روبوتات الدردشة للدعم النفسي.
8. أعتقد أن المجتمع بدأ يتقبل استخدام تطبيقات روبوتات الدردشة في مجال الصحة النفسية.

### ****الكفاءة الذاتية المتصورة****

9. لدي القدرة على الوصول إلى تطبيق أو موقع إلكتروني يتضمن روبوت دردشة للصحة النفسية.
10. أستطيع الحصول على المساعدة إذا واجهت صعوبة أثناء استخدام روبوت الدردشة.

### ****النية السلوكية****

11. إذا توفر روبوت دردشة موثوق يقدم دعماً نفسياً، فسأستخدمه.
12. أنوي استخدام روبوت دردشة ذكي للحصول على الدعم النفسي عند الحاجة.

# **القسم الخامس: العوائق المتصورة**

يرجى تحديد مدى موافقتك على العبارات التالية.

**مقياس الإجابة:**

(1= لا أوافق بشدة /2= لا أوافق/ 3= محايد / 4= أوافق / 5= أوافق بشدة )

13.1 أشعر بالقلق من مشاركة مشاعري الشخصية مع روبوت دردشة.

13.2 أشعر بالقلق من احتمال استخدام بياناتي النفسية بطريقة غير آمنة عند استخدام روبوت الدردشة.

13.3 أعتقد أن روبوتات الدردشة قد لا تقدم دعماً نفسياً بنفس جودة المعالج النفسي.

13.4 أشعر بالقلق من الاعتماد المفرط على روبوتات الدردشة بدلاً من طلب العلاج التقليدي.

## Public Perceptions of Using Mental Health Chatbots in Saudi Arabia

### Introduction

This questionnaire aims to assess the perceptions of the Saudi public regarding the use of **mental health chatbots**.

Completing this questionnaire will take approximately **8 minutes**. Your participation is voluntary. All information you provide will remain confidential and will be used for research purposes only.

☐ **I agree to participate in this study.**

**Section 1. Sociodemographic Characteristics**

**1. Age:**

**2. Gender:**

- ☐ Male
- ☐ Female

**3. Educational level:**

- ☐ Secondary school or below
- ☐ Diploma
- ☐ Bachelor's degree
- ☐ Postgraduate studies

**4. Employment status:**

- ☐ Employee in the mental health sector
- ☐ Employee in the information technology sector
- ☐ Employee in another sector
- ☐ Student
- ☐ Unemployed

**Section 2. Mental Health Background:**

**1. Are you currently experiencing psychological symptoms (such as anxiety, depression, stress, or others)?**

- ☐ Yes
- ☐ No

**2. Have you ever been diagnosed with a mental disorder by a healthcare professional?**

- ☐ Yes
- ☐ No

**3. Have you ever received psychological treatment from a healthcare professional (pharmacological or psychological)?**

- ☐ Yes, I am currently receiving treatment
- ☐ Yes, I received treatment in the past only
- ☐ No, I have never received psychological treatment

**4. During the past 12 months, have you felt that you needed psychological consultation?**

- ☐ Yes
- ☐ No

**Section 3. Prior Use of AI Chatbots**

**1. Have you ever used an intelligent chatbot for psychological purposes, such as emotional support or seeking mental health information?**

- ☐ Yes
- ☐ No

**2. Do you support integrating mental health chatbots into mental health services in Saudi Arabia?**

- ☐ Yes
- ☐ No

**Section 4. Technology Acceptance Constructs (Modified UTAUT Framework):**

Please indicate your level of agreement with the following statements.

**Response scale:**
1 = Strongly disagree 2 = Disagree 3 = Neither agree nor disagree 4 = Agree 5 = Strongly agree

**Perceived Usefulness**

1. I believe that using a chatbot can improve my psychological well-being.
2. A chatbot helps me manage my emotions better.
3. A chatbot may help reduce my stress or anxiety.

**Perceived Ease of Use**

4. It is easy for me to learn how to use AI chat applications for psychological support.
5. Using a chatbot does not require extensive technical expertise.
6. I can easily interact with a chatbot that provides psychological support.

**Social Influence**

7. People around me encourage me to try mental health support chatbots.
8. I believe that society has begun to accept the use of AI chat applications in mental health.

**Perceived Self-Efficacy**

9. I have the ability to access an application or website that includes a mental health chatbot.
10. I can obtain assistance if I encounter difficulties while using a chatbot.

**Behavioral Intention**

11. If a reliable chatbot that provides psychological support is available, I will use it.
12. I intend to use an intelligent chatbot to obtain psychological support when needed.

**Section 5. Perceived Barriers**

Please indicate your level of agreement with the following statements.

**Response scale:**
1 = Strongly disagree 2 = Disagree 3 = Neither agree nor disagree 4 = Agree 5 = Strongly agree

13.1 I am concerned about sharing my personal feelings with a chatbot.

13.2 I am concerned that my psychological data may be used in an unsafe manner when using a chatbot.

13.3 I believe that chatbots may not provide psychological support of the same quality as a human therapist.

13.4 I am concerned about becoming overly dependent on chatbots instead of seeking traditional therapy.
