# Supplementary material for: Behavioral intention to use mental health chatbots in Saudi Arabia: a cross-sectional UTAUT-based study
Source: Front Public Health. 2026 Sep 3;14:1926484. doi: 10.3389/fpubh.2026.1926484 (PMC13582321; doi:10.3389/fpubh.2026.1926484)
Supplement: Supplementary file 2 [file Table_1.docx]

**Supplementary Table S1**. Multivariable Binary Logistic Regression Analysis of Factors Associated with High Behavioral Intention to Use Mental Health Chatbots

| **Predictor** | **B** | **p-value** | **AOR** | **95% CI** |
| --- | --- | --- | --- | --- |
| **Age** | 0.028 | 0.024 | 1.029 | 1.004–1.054 |
| **Gender** | 0.147 | 0.670 | 1.158 | 0.590–2.271 |
| **Educational level (Ref: Secondary school or below)** | — | 0.252 | — | — |
| **Diploma** | −0.935 | 0.084 | 0.393 | 0.136–1.133 |
| **Bachelor's degree** | −0.068 | 0.834 | 0.934 | 0.494–1.767 |
| **Postgraduate studies** | 0.278 | 0.667 | 1.321 | 0.371–4.700 |
| **Current psychological symptoms** | 0.129 | 0.655 | 1.138 | 0.646–2.004 |
| **Needed psychological consultation/support** | 0.609 | 0.032 | 1.839 | 1.053–3.212 |
| **Ever used a mental health chatbot** | 0.100 | 0.733 | 1.105 | 0.622–1.965 |
| **Perceived Usefulness (PU)** | 0.346 | <0.001 | 1.413 | 1.270–1.571 |
| **Perceived Ease of Use (PEOU)** | 0.157 | 0.008 | 1.170 | 1.042–1.314 |
| **Social Influence (SI)** | 0.350 | <0.001 | 1.419 | 1.218–1.653 |
| **Perceived Self-Efficacy (PSE)** | −0.026 | 0.729 | 0.974 | 0.841–1.128 |
| **Perceived Barriers (PB)** | 0.023 | 0.476 | 1.023 | 0.961–1.090 |

PU, PEOU, SI, PSE, and PB were entered as continuous scale scores. The dependent variable was dichotomized behavioral intention (low vs high BI). The model was adjusted for age, gender, education, current psychological symptoms, perceived need for psychological consultation/support, and previous chatbot use. Education was entered as a categorical variable.
